# Supplementary material for: Recombination rate and efficiency of linked selection in small and large stickleback populations
Source: PLoS Genet. 2026 Oct 28;22(10):e1012335. doi: 10.1371/journal.pgen.1012335 (PMC13716167; doi:10.1371/journal.pgen.1012335)
Supplement: Supplementary file 1 [file pgen.1012335.s022.pdf]

Response Letter

Manuscript ID: PGENETICS-D-26-00316
Title: Recombination rate and efficiency of linked selection in small and large stickleback populations

Dear Editor and Reviewers,

We thank the editor and both reviewers for the careful evaluation of our manuscript and for the constructive comments. We are pleased that the reviewers found the study interesting, well-written, and based on a valuable dataset. We have revised the manuscript to address the major concerns raised during review. We have now revised the manuscript following all the comments from the two reviewers, with a specific focus on explaining the limitations and reducing certain assertions. Detailed point-by-point responses are provided below. Reviewer original comments are maintained in black, followed by our responses highlighted in blue.

Reviewer #1

This manuscript uses pedigree-based linkage maps from four freshwater ("small *Ne*") and four marine ("large Ne") nine-spined stickleback populations to ask two questions: (i) whether the classic positive correlation between nucleotide diversity (π) and recombination rate is weaker in small populations, consistent with reduced efficiency of linked selection, and (ii) whether small populations have evolved higher recombination rates, as predicted by theories of recombination modifiers under directional selection. The authors report that the π–recombination relationship is indeed shallower in freshwater populations (and absent in the most homozygous population, PYO), that PYO has significantly higher crossover counts and recombination rate than the marine populations, and that the GWAS suggests a polygenic basis for crossover count variation.

I liked this paper. The questions are important and the dataset is impressive: eight populations, ten high-density linkage maps, and a telomere-to-telomere reference genome, combined with family-based crosses that sidestep the LD-based confounds common in this literature. The biological questions are well-posed and the overall conclusions point in the right direction.

That said, several aspects of the analysis and interpretation need to be addressed before the headline claims can be accepted. In particular the claim of adaptive evolution of recombination rate in PYO and the polygenic basis of recombination rate variation. My concerns fall into three areas: (1) the two Ne estimators speak to very different timescales, and the authors' interpretation does not adequately distinguis h between them; (2) the "elevated recombination in small populations" signal is effectively an n = 1 inference (PYO) that is also the population most vulnerable to technical inflation of map length; and (3) the nucleotide-diversity and GWAS analyses have methodological choices that should be revisited. Importantly, because this paper is well motivated by theory and the data set itself stands on it own, I do not think these changes

(even though they will likely result in the result becoming "negative") would change the overall scientific value or impact of this work in any sense. Individual points follow.

Response:

We thank Reviewer #1 for the thoughtful and constructive review. We appreciate the positive assessment of the motivation, dataset, and overall value of the study. We have addressed these concerns through new analyses and substantial revisions to the framing of the Results and Discussion.

Major comment 1

The crossover–Ne correlation is being interpreted more strongly than the data support, because the two Ne estimators employed by the authors measure different things. The paper reports a significant negative relationship between crossover count and Ne estimated by MSMC2, but not with CurrentNe2 ($p = 0.098$; lines 183–184). MSMC2's harmonic-mean Ne over 2–200 kya substantially predates the ~10 kya freshwater colonization that the authors themselves invoke on line 361. For the freshwater populations, the MSMC2-hm therefore largely reflects marine-ancestral demography, not the small-population conditions that are supposed to have driven recombination rate evolution. The negative crossover–MSMC2 relationship cannot be read as evidence that recombination has evolved in response to current drift, and the non-significance of the CurrentNe2 relationship (which is the Ne metric that actually bears on the authors' hypothesis) deserves considerably more attention. The current framing in the Abstract, Results and Discussion reads as if both estimators tell the same story; they do not. I think the authors need to (a) state explicitly what timescale MSMC2 covers and what it therefore measures for each ecotype, (b) center the evolutionary interpretation on CurrentNe2 (or on a measure tied to post-colonization drift, e.g. ROH load or current π), and (c) soften or remove language that implies a general Ne effect when only the pre-colonization estimator is significant.

Response:

We thank the reviewer for this insightful suggestion about our demographic timescale. We completely agree that MSMC2 and CurrentNe2 capture distinct evolutionary windows, and our MSMC2 estimate should have been based on the post-colonization freshwater constraints.

To address this, we re-calculated the historical N_e by using the N_e at 10kya. Interestingly, the negative correlation between crossover count and this post-colonization N_e remains statistically significant ($p=0.02$), while the correlation with CurrentNe2 is non-significant ($p=0.09$). The discrepancy between the historical $\mathrm{N_e}$ and contemporary N_e could be due to the evolutionary time lag, and the selection on the modifiers of the recombination landscapes evolves over deep evolutionary time. Recent, rapid fluctuations in modern population size alter CurrentNe estimates immediately, but have occurred far too quickly for the biological machinery controlling crossover

numbers to adapt or catch up.

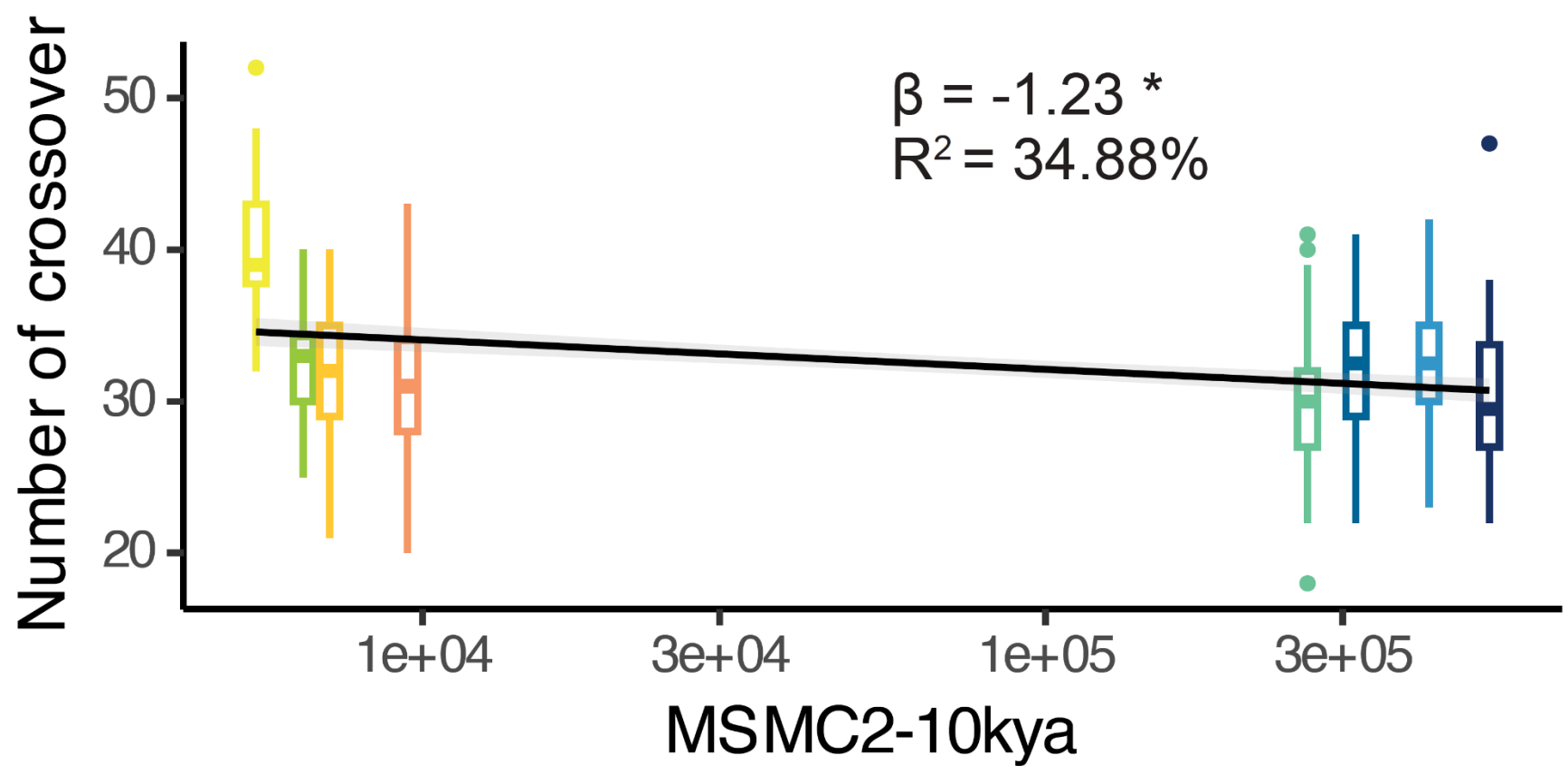

However, we have identified an important caveat that the smallest population, PYO, heavily drove the significant relationship. This aligns with the reviewer's call for a more cautious interpretation. While we transparently reported this caveat throughout the manuscript, we have now softened our language about the N_e effect on recombination rates. Please see our response to Major comment 2.

Major comment 2

The claim of "elevated recombination rate in small populations" rests on a single population. Yet, the freshwater–marine rate difference disappears when PYO is excluded (lines 197–198), the GLMM correlation in Figure 2C is visibly driven by that one point, and the "adaptive evolution of recombination" narrative in the Discussion and Abstract rests essentially on PYO alone. I have a few suggestions for the authors to remedy this issue.

Figure S12 already reports the ecotype comparison of sex-averaged recombination rate and per-individual crossover counts with PYO excluded; this analysis should be brought into the main text and Figure 2, and the text should state clearly that the crossover–Ne signal hinges on this one population.

PYO is a technical outlier in ways that matter for map-length inference: it is the most homozygous population (≈91% of its genome in ROH, line 375), has by far the lowest marker count in its linkage map (158,980, vs. ~3.8 M in the marine maps), and simultaneously has the highest estimated genetic map length (1,815 cM; Table 2). A sparse marker set combined with residual genotyping error is a known cause of map-length inflation in Lep-MAP3. The authors use ROH to argue that recombination in freshwater populations is probably underestimated (lines 376–378), but this logic cuts against the inference of an elevated rate in PYO specifically: if ROH plus sparse markers depress recombination estimates in the other three freshwater populations, why should PYO be exempt, given that it has more ROH and fewer markers than any of them? A sensitivity analysis, e.g., re-estimating map length after down-sampling the marine maps to match PYO's informative-marker density, masking simulated ROH tracts, or both, would help establish whether the PYO signal is biological or methodological. It would also be helpful to report the

number of putative double crossovers in close physical proximity, which is a useful marker of genotyping-error-driven map inflation.

With only one point in the relevant small-Ne region of the parameter space, the “elevated recombination rate evolves in small populations” framing should be presented as a hypothesis generated by these data, not a conclusion supported by them.

Response:

We thank the reviewer, and we agree that the elevated recombination rate in freshwater populations is driven mainly by a single PYO population and that this needs to be made explicit. To test the PYO recombination rate estimate, we downsampled the SNP markers in the other seven populations to match the number and approximate genomic distribution of PYO's informative markers (~158,980), rebuilt linkage maps using Lep-MAP3, and replicated the analyses 100 times for each population. The results are summarised here:

**Population**	**Full SNPs recombination rate**	**Downsampled to PYO SNP density-level rate (mean of 100 reps; 95% CI)**
KRK	3.90	3.96 (3.91–4.01)
POR	3.81	3.86 (3.83–3.92)
TVA	3.85	3.85 (3.81–3.89)
BYN	3.76	3.79 (3.76–3.83)
RYT	3.71	3.75 (3.72–3.79)
UME	3.68	3.74 (3.69–3.78)
RAA	3.64	3.68 (3.65–3.73)
PYO	4.24	-

Downsampling all other seven populations to PYO's marker density raised their estimated change by only 0.05 cM/Mb on average, and the estimates are still well below PYO’s observation of 4.24 cM/Mb. The influence of marker density is therefore far too small to explain PYO's ~0.3–0.5 cM/Mb elevation, indicating that the PYO signal is biological rather than a marker-density artefact. These results and methods have been added to the main text, and the above table is added as Supplementary Table 4. We also note PYO's small N_e and high homozygosity rate are independently corroborated by other studies of the same populations (high FROH in Chen et al. 2025; contemporary N_e in Feng et al. 2025), so the population's outlier status with respect to N_e is well established and not an artefact of the present linkage map.

We did not mask ROH as an additional sensitivity analysis because pedigree-based linkage maps use only informative markers that are heterozygous in the parent and segregate in the offspring.

As discussed in Ln 385-393, the absence of elevated recombination rates in the other populations may stem from the interplay between selection and genetic drift. In small populations, inflated genetic drift often overpowers the efficacy of selection, preventing the accumulation of high-recombination modifiers. However, the PYO population appears to represent a critical exception where an urgent adaptive demand for elevated recombination (likely to purge accumulated genetic load) overcame the effects of drift. While testing this hypothesis across additional small populations remains an ideal future direction, PYO stands as the most extreme bottlenecked population identified across all our prior studies of nine-spined sticklebacks, making it a unique and invaluable case study. We have now reframed the discussion to make it more digestible.

Figure 2 has now been modified according to the above two comments, where the historical N_es have been changed to those at 10kya. Instead of incorporating Figure S12 in Figure 2, we added the letters to indicate significant differences between PYO and the other populations based on a post-hoc Tukey's HSD test ($p < 0.05$) in Figure 2B.

References

Chen, Y., Feng, X., Reid, K., Zhang, C., Löytynoja, A., & Merilä, J. 2025. Dynamics of deleterious mutations and purifying selection in small population isolates. *Molecular Biology and Evolution*, 42(7), msaf110.

Feng, X., Löytynoja, A., & Merilä, J. 2025. Estimating recent and historical effective population size of marine and freshwater sticklebacks. *Molecular Ecology*.

Major comment 3

Nucleotide diversity is estimated without invariant sites, and this could distort the central result. π is estimated with VCFtools on SNPs only (line 494). VCFtools does not account for missing data or for invariant but callable sites, and estimating π this way biases the result, particularly when comparing populations that differ in per-site callability. This is almost certainly the case between the marine parents (very high marker density) and freshwater parents (far fewer informative markers and extensive ROH). Because the π–recombination relationship and the ecotype contrast in π are central results, this matters. I think the authors need to re-estimate π with a method that uses invariant sites and handles missingness explicitly (e.g. pixy) and confirm that (i) the positive π–recombination correlation, (ii) its shallower slope in freshwater, and (iii) the absence of correlation in PYO are all robust to this change. The authors should also report the per-population callable fraction and pairwise differences in callability so the reader can judge the magnitude of the potential bias.

Response:

Thank you for this comment. We agree, and we therefore reestimated nucleotide diversity using Pixy while retaining the same 1 Mb windows and 200 kb sliding intervals used in the original analysis. We found that estimates of π from Pixy and VCFtools were highly consistent across

populations, with Pearson correlation coefficients ranging from 0.848 to 0.956. When applying the newly estimated per-window π, the main results remained unchanged - π was positively correlated with recombination rate in all populations except PYO, even accounting for genotyping rate as a covariate (see the second table below). Specifically, PYO was non-significant in the association test β_1 = -1.16E-06, P = 0.196, and this remained true after including callable fraction as a covariate, β_1 = -6.48E-07, P = 0.464. Thus, although the reviewer's concern is well founded, our conclusions are robust to the method used to estimate nucleotide diversity.

**Population**	**Pearson correlation between Pixy and VCFtools**
BYN	0.950
KRK	0.956
POR	0.953
PYO	0.848
RAA	0.950
RYT	0.926
TVA	0.954
UME	0.948

Association test between Pixy π and crossover number

**Population**	β_1	β_0	R^2
RAA	6.98E-05***	2.40E-03***	21.39%
TVA	7.50E-05***	2.32E-03***	23.55%
POR	6.39E-05***	2.35E-03***	22.06%
UME	8.09E-05***	2.07E-03***	28.42%
KRK	1.63E-05***	5.25E-04***	8.87%
BYN	2.89E-05***	6.76E-04***	21.52%
RYT	2.46E-05***	7.28E-04***	15.45%
PYO	-1.16E-06	1.64E-04***	0.09%

Major comment 4

The Methods for MSMC2 and CurrentNe2 are deferred to Zhang et al. (2026), a submitted manuscript that is not accessible to the reader (line 506). Even with the Supplementary Materials and Methods in hand, the reader cannot reconstruct these Ne estimates: the mutation rate and generation time assumed for MSMC2, the segment/bin filters, the CurrentNe2 input SNP set and assumed recombination rate, and the minor allele frequency thresholds are all missing. At resubmission, please provide a self-contained Methods description of MSMC2 and CurrentNe2 that does not depend on an unpublished paper.

Response:

Thank you for bringing up this concern. This manuscript has now been made accessible in *BioRxiv* (doi: https://doi.org/10.64898/2026.07.31.741990) for your reference. We have also added a section about the N_e estimation in the supplementary methods of this manuscript.

Major comment 5

Lines 498–500 state that windows with >20% repeat content were excluded from gene-density and CpG estimation. It is not stated whether this filter was also applied to the π and recombination-rate window analyses. Because repeat content is strongly non-randomly distributed with respect to recombination rate (enriched at telomeres and centromeres), inconsistent masking across analyses could either manufacture or obscure associations. Please state explicitly which analyses used the >20% filter, report how many 1 Mb windows were removed per chromosome, and confirm that the main results are insensitive to alternative repeat-content thresholds (e.g., 10%, 30%, or a gradient).

Response:

Thanks. We can confirm that we applied the >20% filter in both π and recombination rate window analyses. 28.38% of the windows were removed in total. This has now been explicitly stated at Ln 529. We have also provided the raw tables, including the number of retained and excluded windows for each chromosome, for the reviewer's reference.

Major comment 6

The GWAS is run on the wild-caught F0 parents — a total of 80 individuals (Table 1) distributed across eight populations. This is modest power for mapping a quantitative trait with a genome-wide test. Across the three methods, only three QTL survive FDR < 0.05 in FarmCPU and GLM, and zero survive in MLM. The "48 suggestive QTL" from FarmCPU are based on a nominal p < 1e-05 cutoff without genome-wide correction, and the between-method concordance (MLM 4, GLM 48, FarmCPU 48) is not reassuring. These data do not support the current

wording of "GWAS analyses suggested a polygenic basis for recombination rates" (Abstract) and especially not the Author Summary statement that "recombination is not controlled by a single gene but is a complex trait influenced by many genes in different parts of the genome." I think the authors should (a) report QQ plots, λGC for all three methods (Manhattan plots are already in Figure S10 of the supplement); (b) report the per-method set overlap of significant hits; (c) state the sample size and expected statistical power; and (d) soften the polygenicity language to reflect the FDR-corrected results.

Response:

We agree with this comment and have added the QQ plot and the genomic inflation factors for all three GWAS models. These results are now presented in Figure S11. The λGC values from all methods were close to 1, indicating no substantial inflation of the test statistics. We also modified the wording throughout the manuscript and softened the language with respect to polygenicity. The grey shaded area represents the 95% confidence envelope expected under the null hypothesis of no association. FarmCPU terminated after the first iteration, which is equivalent to the GLM before pseudo-QTNs are incorporated; therefore, FarmCPU and GLM p-values were identical or nearly identical, and the GLM points are largely hidden underneath the FarmCPU points.

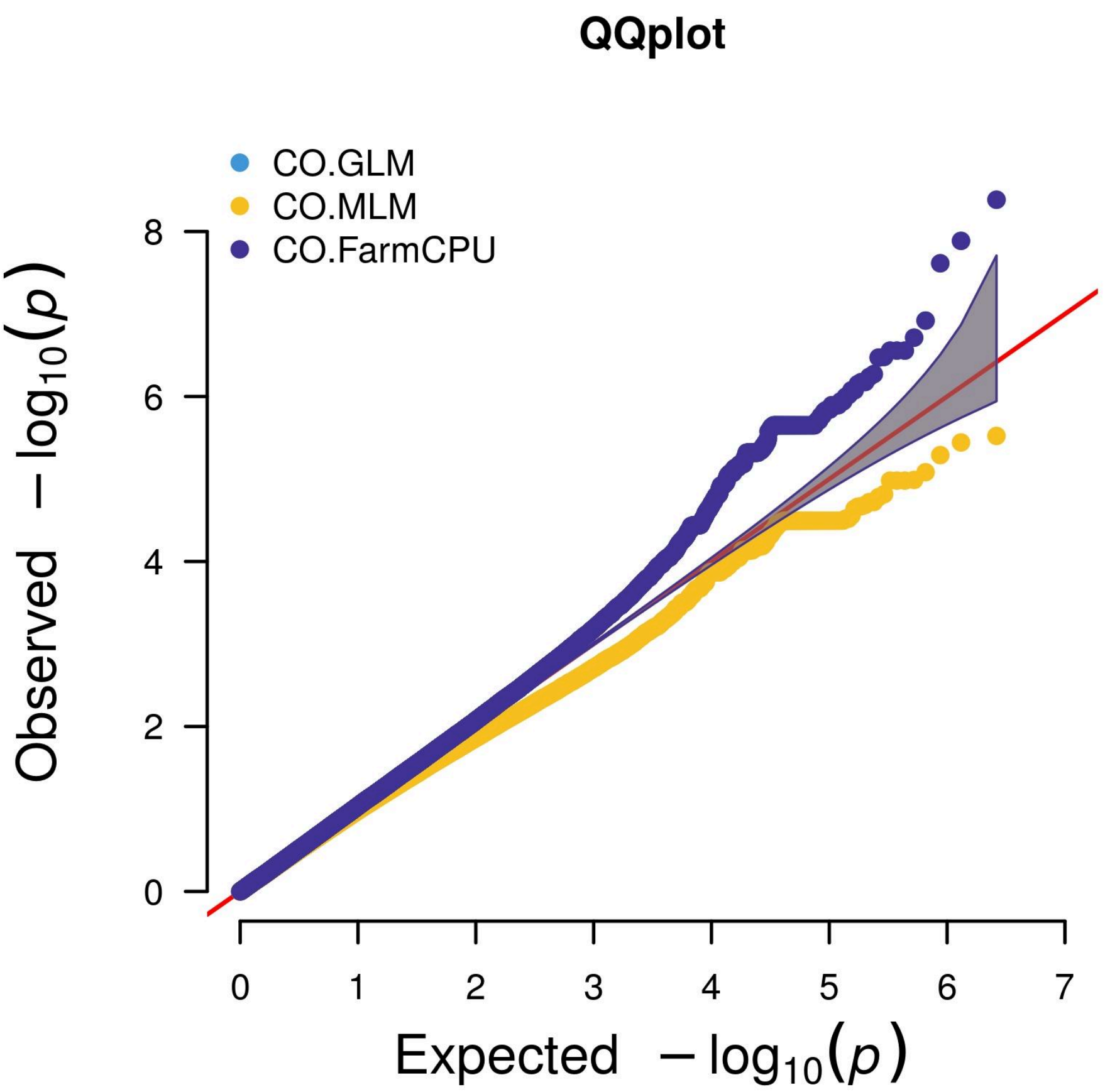

Major comment 7

The Ne values reported in the Abstract do not match Table 1. The Abstract gives mean Ne ≈ 2,578 for freshwater and 86,742 for marine populations, but neither value corresponds to the mean of either Ne estimator in Table 1. For the marine populations, the mean of NeMSMC2-hm is ≈264,925 and the mean of NeCurrentNe2 is ≈14,324. I cannot reconstruct how 2,578 or 86,742 were obtained from Table 1. Please state in the Abstract, Table 1 legend, and Methods exactly what Ne estimator and summary statistic are being reported, and reconcile the numbers. A range across the four populations of each ecotype would perhaps be more informative than a point estimate.

Response:

Thank you for pointing out this error. The N_es in the abstract were from a previous version of the calculation, which should have been changed. The numbers have now been updated according to the first major comment to 7,096 and 376,828 for freshwater and marine populations, and they were added to results at Ln167 and Ln168.

Minor comments

Minor comment 9

Lines 561–563 duplicate lines 558–559 almost verbatim on FarmCPU controlling for population stratification. Please remove the duplicate. Similar near-duplicate phrasing also appears in the Discussion.

Response: Corrected.

Minor comment 10

Line 417 cites “Zhang et al. 2025” whereas every other citation of that reference in the manuscript is to “Zhang et al. 2026” (e.g., lines 121, 402, 497, 506, 512).

Response: Corrected. The citation now consistently reads Zhang et al. 2026.

Minor comment 11

Hotspot (top 5% of windows) and coldspot (zero crossovers) are operational cutoffs, not biological categories, and are sensitive to map resolution. Given mean per-chromosome crossover counts of ~1.84 per meiosis, a 1 Mb window with zero observed crossovers is often a sampling artifact rather than a biological cold spot. Please either demonstrate robustness to alternative cutoffs (e.g., top 1% / 10%) or frame the dichotomy as operational.

Response:

We thank the reviewer for highlighting the importance of threshold robustness. When establishing our filter thresholds, we found that the entire bottom 5% of the genomic distribution consists of regions with zero recombination rates. Consequently, our coldspots are not defined by an arbitrary operational cutoff, but rather represent absolute biological "recombination deserts".

Regarding the hotspots, to ensure overall robustness, we tested alternative operational thresholds for hotspots, including the top 5%, 10% and 15%. The hotspots are still located at the chromosomal ends mostly, and the downstream comparison of CpG density yielded qualitatively identical results across all thresholds with significant distinctions between coldspots and hotspots. We have clarified this in the revised manuscript at Ln 523

Minor comment 12

Figure S9 already reports inversion genotype frequencies across populations and per-genotype recombination on chr19. Please reference Figure S9 when the chr19 inversion is raised in the Discussion, and state explicitly in the main text whether the inversion region was included in or masked from the genome-wide rate estimates.

Response:

Thank you for this comment. We have now quoted Figure S9 in the Discussion at Ln 411, and this region was included in all the downstream analyses because it displays polymorphism in allelic frequencies. We have now reflected it in Ln 505 in the Methods.

Minor comment 13

Table 1 legend defines NeMSMC2-hm as the harmonic mean over 2–200 kya, but the main text introduces "past Ne estimated by MSMC2" (line 183) without this timescale. Please state the timescale the first time the estimator is used in the text.

Response:

Thank you for pointing this out. We have now added the time details in the main text.

Minor comment 14

Please ensure the GitHub repository (https://github.com/LeoHongboWANG/Recombination_in_Sticklebacks) includes a README describing script dependencies, input-file expectations, and how to reproduce the main figures. I did not evaluate the code.

Response:

We have updated the GitHub repository to include a README file describing software dependencies, input file formats, expected directory structure, and commands for reproducing the localized recombination rate calculations and main figure inputs(https://github.com/LeoHongboWANG/Recombination_in_Sticklebacks/).

Minor comment 15

Typos: line 121–122 “To enhance the accuracy genome-wide…” is missing “of”; line 72 has an extra space before the period; line 399 refers to “methylation levels” where “CpG content” is what is actually measured; I think “Varandharjan” on line 480 is a misspelling of Varadharajan.

Response: Corrected. We fixed the missing “of”, removed the extra space, replaced “methylation levels” with “CpG content”, and corrected “Varandharjan” to “Varadharajan”.

Response to Reviewer #2

I thought this manuscript was interesting and well-written. Few studies have examined patterns of variation in recombination rate for multiple wild populations of the same species, so this study is very useful. The paper also provides interesting insight into the relationship between nucleotide diversity, recombination rate and effective population. Lastly, It identifies candidate genes/regions contributing to variance in recombination rate.

We thank Reviewer #2 for the positive assessment of the manuscript and for highlighting two important issues: the possibility that the elevated recombination rate in PYO could reflect technical artifacts associated with lower marker density, and the possibility that freshwater–marine differences in recombination estimates could be influenced by genome size or structural differences. We have addressed these points through additional analyses and by revising the interpretation of our results.

Comment 16

The first was with the emphasis that the PYO population had the smallest Ne and the highest recombination rate. Given that this population appeared as an outlier it was not reassuring that it had the lowest quality linkage map, with substantially fewer markers. Could the recombination rate be inflated by the low marker density? Could you down sample the other populations to the same density to ensure it is not an artefact? Were there also fewer markers for this population for the estimation of Ne? If so, again could this be driving the pattern? A considerable fraction of the discussion and angle of the paper is focused on this exceptional (outlier) population so I think there needs to be more reassurance in the validity of this result.

Response:

Thank you for bringing up this concern. We agree that PYO required additional scrutiny because it had both the highest estimated recombination rate and the lowest number of informative linkage-map markers. To test whether the PYO recombination estimate could be inflated by lower marker density, we performed a marker-density downsampling analysis. Specifically, we downsampled the marker sets of the other populations to match the number and approximate genomic distribution of informative markers in PYO and reconstructed linkage maps using the same Lep-MAP3 pipeline. This analysis was repeated 100 times per population. Please see our response to Comment 2 above.

We also clarified that the N_e estimates were not obtained from the linkage-map marker sets. Instead, MSMC2 and CurrentNe2 estimates were based on whole-genome sequence data from wild-caught individuals, using the filtering and callable-site criteria now described in the Methods. Therefore, the lower number of informative linkage-map markers in PYO does not directly determine its N_e estimate. However, we agree that low diversity and high homozygosity can influence both demographic inference and recombination detection, and we refer readers to the caveat discussed at Ln453.

Finally, we emphasize that PYO represents a pond population outlier. Even though the downsampling analysis supports the robustness of the rebuilt linkage maps and the estimated recombination rates, we avoid using PYO alone as evidence for a general association between small N_e and elevated recombination.

Comment 17

The second is that the observed recombination rate difference between the ecotypes could be due to differences in genome size and structure differences. This is a fairly large caveat and should be more thoroughly addressed. Is there a freshwater reference that could be used to check for this potential effect? Is there another way to address this potentially fundamental constraint of the current dataset?

Response:

Thank you for this comment. Yes, we agree that differences in genome size could influence recombination rate estimates expressed in cM/Mb, especially because physical distances were measured relative to a marine reference genome. In this and earlier version of the manuscript we explicitly acknowledged this. However, this “caveat” makes our finding of higher recombination rate in freshwater population(s) conservative: it would tend to underestimate freshwater populations’ recombination rates.

In detail, any bias introduced by our method is conservative when comparing recombination rates between freshwater and marine populations. The marine reference genome used here is larger than the freshwater genome: the TVA genome is 429,327,121 bp (GenBank: GCA_056822235.1) excluding sex chromosomes, whereas the PYO genome (GenBank: GCA_902500615.4) is 406,173,618 bp, or 409,127,584 bp when unassembled contigs are included. These contig sequences may include insertions, deletions, or larger structural variants. Although we discuss the

composition of the unassembled contigs in detail in our telomere-to-telomere genome study (*BioRxiv* doi: https://doi.org/10.64898/2026.07.31.741990), including them when estimating freshwater chromosome length would inflate the physical genome size and therefore underestimate the freshwater recombination rate measured in cM/Mb. Conversely, using the pond genome as the reference would increase the estimated recombination rate for PYO. Thus, the higher recombination rate observed in the freshwater population cannot be explained simply by differences in genome size. If anything, accounting for the smaller freshwater genome would likely increase the estimated difference in recombination rate between freshwater and marine populations.

At present, our T2T reference genome available for this analysis is from a marine population. Although a freshwater reference assembly is available, it is not of comparable T2T quality and therefore we cannot yet fully resolve whether genome-size or structural differences contribute to the freshwater and marine differences in cM/Mb estimates. We previously used the PYO genome assembly to estimate the PYO recombination rate and its recombination rate was estimated to be 4.4 cM/Mb, not too far away from what we estimated using the marine (TVA) genome in this study (4.2 cM/Mb). For this reason, we now place greater emphasis on crossover counts, which are less directly affected by reference-based physical distance scaling (discussed at Ln538), and we interpret cM/Mb differences between ecotypes with caution (Ln457-458).
