# Supplementary material for: Recombination rate and efficiency of linked selection in small and large stickleback populations
Source: PLoS Genet. 2026 Oct 28;22(10):e1012335. doi: 10.1371/journal.pgen.1012335 (PMC13716167; doi:10.1371/journal.pgen.1012335)
Supplement: Supplementary file 2 [file pgen.1012335.s001.docx]

### **S1 Text. Supplementary material and methods**

#### *Sampling and generation of crosses*

As in Zhang et al. 2025, the same families from eight different *P. pungitius* populations were used in this study (Figure 1A, Table 1). Four of these were sampled from isolated freshwater ponds in Finland and Sweden, which were known to have small effective population sizes (*N_e_*), while the other four were obtained from outbred marine populations in the Baltic Sea of larger *N_e_* (Table 1). The parental fish from these populations were collected with beach-seine nets or minnow traps between May-June 2018 and transported alive to aquaculture facilities at the University of Helsinki. The fish from different populations were maintained separately in 1m^3^ plastic tanks with flow-through freshwater, and they were fed twice a day *ad libitum* with frozen chironomid larvae until used in artificial crosses.

Within each population, five full- or half-sib families were created through artificial fertilisations by randomly pairing wild-caught individuals (Fraimout et al. 2022). Most of these crosses were two generational (i.e. parental and F_1_ generation) while some were three generational including a F_2_ generation. In total, the final dataset included 80 parents, 364 parent-offspring trios and 734 meioses. More details for how the crosses were generated, please refer to Zhang et al. 2025. After the rearing period, all fish were over-anesthetized as described above, and the fin clips were preserved in 95% alcohol for subsequent DNA extraction.

#### *DNA extractions and sequencing*

Following the salting-out method of Sunnucks and Hales (1996), genomic DNA was extracted from the ethanol-preserved fin clips of offspring and parents by applying the DNeasy Blood & Tissue Kit. The concentration and purity of the extracted DNA were assessed using a NanoDrop spectrophotometer and an Invitrogen Qubit 4.0 instrument. DNA was available for four to five families from each population, totalling in 80 parents and 364 offspring (Table 1). In addition to the DNA data for TVA and POR populations as reported in Zhang et al. (2023), DNA samples from all remaining specimens were submitted to the Beijing Genomics Institute (BGI) where PCR-free library construction was performed using their proprietary DNBseq platform for next-generation sequencing. All samples underwent whole-genome resequencing with a target coverage of 35×.

#### *Read mapping and SNP calling*

Paired-end short-read data were mapped to the NSPV9_T2T assembly of the nine-spined stickleback (submitted manuscript). To prevent mismapping, the pseudoautosomal region was included only once in chrX, and chrY was excluded from the reference genome during read mapping for female samples. The resulting BAM files were sorted using SAMtools (v1.18; Li et al. 2009), and PCR duplicates were marked using Picard MarkDuplicates (v2.18; http://picard.sourceforge.net). Variant detection was carried out with GATK HaplotypeCaller (v4.3.0.0; Poplin et al. 2017) in ERC mode, followed by base quality score recalibration (BQSR) and hard filtering, following GATK best practices (DePristo et al. 2011). The gVCF files generated for each sample were combined and jointly genotyped across populations using GATK’s CombineGVCFs and GenotypeGVCFs modules. Specifically, variants located within the sex determination region on chrX (1-18.875Mb) were called separately for samples of the two sexes, with ploidy set to 2 for females and 1 for males.

#### *Identification of inversion genotypes in population samples*

An assessment of the crossover count was conducted to the reported inversion region on chr19 (~5.8-7.2Mb; Varadharajan el al. 2019). The inversion genotypes of the parents (i.e., F_1_ parents in three-generation pedigrees and F_0_ parents in two-generation pedigrees) were determined using principal component analysis (PCA) in PLINK (v1.90; Chang et al. 2015), based on SNP variants within the inversion region. Parents were grouped into three clusters, and each represented one genotype (0/0: ancestral state homozygote; 0/1: heterozygotes; 1/1: inversion homozygote). The crossover counts obtained from the pedigree-level linkage maps were examined separately for these three groups of parents.

#### *Estimate effective population sizes (N_e_)*

To evaluate demographic trends across distinct evolutionary timescales, we estimated both historical and contemporary effective population sizes (*N_e_*). Historical *N_e_* trajectories were reconstructed using Multiple Sequentially Markovian Coalescence (MSMC2; Schiffels & Wang 2020). For each population, we used data from two tros to construct phased genomic datasets. To account for variations in sequence accessibility and prevent artifacts from poorly mapped genomic regions, a genome-wide mappability mask was generated using snpable2 (<https://sourceforge.net/u/lh3lh3/profile/>). Variants were called across chromosomes using *bcftools mpileup*, and the variants were phased using read-backed phasing via WhatsHap (v2.0; Martin et al. 2016). The MSMC2 parameters were configured with a maximum of 100 iterations (-i 100), an upper time bound for the most recent common ancestor of 16 (-t 16), and an atomic time interval pattern (-p) of '1 * 2 + 25 * 1 + 1 * 2 + 1 * 3'. To scale the demographic trajectories to absolute time, we applied the real average parental age of each population as the generation time for each population, alongside the mutation rates estimated from Zhang et al. (2026). We extracted the *N_e_* exclusively at the post-colonization time after last glaciation at 10kya.

Additionally, contemporary *N_e_* was estimated using CurrentNe2 (Santiago et al. 2025). This approach leverages linkage disequilibrium (LD) patterns between SNPs. To account for regional population substructure, we allowed for migration between populations by incorporating a migration parameter (-x). Input VCF files were thinned using VCFtools (--thin 10; v0.1.16; Danecek et al. 2011) to retain a maximum of one SNP per 10 base pairs, ensuring a computationally efficient dataset of fewer than 2 million SNPs for CurrentNe2. Population-specific recombination rates were derived from empirical genetic maps generated in this study.

#### *References*

Chang CC, Chow CC, Tellier LCAM, Vattikuti S, Purcell SM, Lee JJ. 2015. Second-generation PLINK: rising to the challenge of larger and richer datasets. *GigaScience* 4:7.

Danecek P, Auton A, Abecasis G, Albers CA, Banks E, DePristo MA, Handsaker RE, Lunter G, Marth GT, Sherry ST, et al. 2011. The variant call format and VCFtools. Bioinformatics 27:2156–2158.

DePristo M, Banks E, Poplin R, Garimella K, Maguire J, Hartl C, Philippakis A, del Angel G, Rivas MA, Hanna M, McKenna A, Fennell T, Kernytsky A, Sivachenko A, Cibulskis K, Gabriel S, Altshuler D, Daly M. 2011. A framework for variation discovery and genotyping using next-generation DNA sequencing data. *Nat Genet.* 43:491-498.

Fraimout A, Päiviö E, Merilä J. 2022. Relaxed risk of predation drives parallel evolution of stickleback behaviour. *Evolution* 76: 2712-2723.

Li H, Handsaker B, Wysoker A, Fennell T, Ruan J, Homer N, Marth G, Abecasis G, Durbin R, 1000 Genome Project Data Processing Subgroup. 2009. The sequence alignment/map (SAM) format and SAMtools. *Bioinformatics* 25:2078–2079

Martin M, Patterson M, Garg S, Fischer SO, Pisanti N, Klau GW, Schoenhuth A, Marschall T. 2016. WhatsHap: fast and accurate read-based phasing. *bioRxiv*. doi: 10.1101/085050

Poplin R, Ruano-Rubio V, DePristo MA, Fennell TJ, Carneiro MO, Van der Auwera GA, Kling DE, Gauthier LD, Levy-Moonshine A, Roazen D, Shakir K, Thibault J, Chandran S, Whelan C, Lek M, Gabriel S, Daly MJ, Neale B, MacArthur DG, Banks E. 2017. Scaling accurate genetic variant discovery to tens of thousands of samples. *bioRxiv*. doi: 10.1101/201178.

Santiago E, Köpke C, Caballero A. 2025. Accounting for population structure and data quality in demographic inference with linkage disequilibrium methods. *Nat. Commun.* 16:6054.

Schiffels S, Wang K. 2020. MSMC and MSMC2: The Multiple Sequentially Markovian Coalescent. *Methods Mol Biol.* 2090:147-166.

Sunnucks P, Hales DF. 1996. Numerous transposed sequences of mitochondrial cytochrome oxidase I-II in aphids of the genus *Sitobion* (Hemiptera: Aphididae). *Mol Biol Evol.* 13:510-524.

Varadharajan S, Rastas P, Löytynoja A, Matschiner M, Calboli FC, Guo B, Nederbragt AJ, Jakobsen KS, Merilä J. 2019. A high-quality assembly of the nine-spined stickleback (Pungitius pungitius) genome. *Genome Biol Evol.* 11(11):3291-3308.

Zhang C, Reid K, Sands AF, Fraimout A, Schierup MH, Merilä J. 2023. De novo mutation rates in sticklebacks. *Mol Biol Evol.* 40:msad192.

Zhang C, Wang H, Lv L, Reid K, Wang D, Schierup MH, Merilä J. 2026. Intra-specific evidence supports drift-barrier hypothesis of mutation rate evolution. Submitted manuscript.
