# Supplementary material for: Recombination rate and efficiency of linked selection in small and large stickleback populations
Source: PLoS Genet. 2026 Oct 28;22(10):e1012335. doi: 10.1371/journal.pgen.1012335 (PMC13716167; doi:10.1371/journal.pgen.1012335)
Supplement: Supplementary file 3 [file pgen.1012335.s002.docx]

### **S1 Table. Average per-chromosome crossover numbers for the eight populations.** Populations shaded in blue represent the marine populations, while those in yellow represent freshwater counterparts. (Chr: chromosome)
