# Supplementary material for: Recombination rate and efficiency of linked selection in small and large stickleback populations
Source: PLoS Genet. 2026 Oct 28;22(10):e1012335. doi: 10.1371/journal.pgen.1012335 (PMC13716167; doi:10.1371/journal.pgen.1012335)
Supplement: Supplementary file 4 [file pgen.1012335.s003.docx]

### **S2 Table. Summary of the best multivariate models influencing localised sex-averaged (above) and and sex-specific (below) recombination rates** (Significance codes: <0.001: ***, <0.01: **, < 0.05: *).

A. Sex-averaged recombination rate (all are scaled values, Gaussian distributed model in glmmTMB)

|  |  | **Population-level** | | **Ecotype-level** | | | |  |
| --- | --- | --- | --- | --- | --- | --- | --- | --- |
|  |  | Slope (β) | Variance explained | Slope (β) | | Variance explained | |  |
| Fixed main effect | CpG content | 0.52*** | 27.44% | | 0.62*** | | 38.65% | |
| Random effect | Population |  | 0.001% | |  | |  | |
| Intercept |  | -4.3e-08 |  | | -2.7e-06 | |  | |
|  |  |  |  |  |  |  |  |  |

B.	Sex-specific recombination rate (negative binomial model in glmmTMB)

|  |  | **Population-level** | | **Ecotype-level** | |
| --- | --- | --- | --- | --- | --- |
|  |  | Slope (β) | Variance explained | Slope (β) | Variance explained |
| Fixed main effect | CpG | 37.10*** | 17.00% | 62.85*** | 27.90% |
|  | Ecotype | -0.33* | 0.27% |  |  |
|  | Gene density | -0.37** | 0.0046% | -0.45 | 0.00% |
|  | Sex | -1.84*** | 9.42% | -3.28*** | 16.67% |
| Fixed interaction terms | Ecotype:CpG | 8.31 | 0.074% |  |  |
|  | CpG:Sex. | 34.43*** | 5.38% | 66.87*** | 6.19% |
|  | Gene density:Sex | 0.73** | 0.00% | 1.12** | 0.45% |
| Random effect | Population |  | 0.19% |  |  |
| Intercept |  | 1.29*** |  | 0.36 |  |
