# Supplementary material for: Recombination rate and efficiency of linked selection in small and large stickleback populations
Source: PLoS Genet. 2026 Oct 28;22(10):e1012335. doi: 10.1371/journal.pgen.1012335 (PMC13716167; doi:10.1371/journal.pgen.1012335)
Supplement: Supplementary file 5 [file pgen.1012335.s004.docx]

### **S3 Table. Pedigree information for each population.** Pedigree type codes 1-3, respectively, refer to the grey, purple, and light purple pedigrees in Figure 1B.

| **Ecotype** | **Population** | **Pedigree** | **Offspring_num** | **Pedigree_type** |
| --- | --- | --- | --- | --- |
| Marine | TVA | TVA24 | 10 | 3 |
|  |  | TVA32 | 10 | 2 |
|  |  | TVA34 | 10 | 2 |
|  |  | TVA36 | 10 | 2 |
|  |  | TVA38 | 10 | 2 |
|  | POR | POR02 | 10 | 2 |
|  |  | POR06_12_13_1 | 10 | 1 |
|  |  | POR06_12_13_2 | 10 | 1 |
|  |  | POR11 | 10 | 2 |
|  |  | POR31 | 10 | 2 |
|  | UME | UME05_06 | 10 | 1 |
|  |  | UME14_34_1 | 10 | 1 |
|  |  | UME14_34_2 | 10 | 3 |
|  |  | UME18 | 10 | 3 |
|  |  | UME22 | 10 | 3 |
|  | RAA | RAA04 | 10 | 2 |
|  |  | RAA06_09 | 10 | 1 |
|  |  | RAA13 | 10 | 2 |
|  |  | RAA14 | 10 | 2 |
|  |  | RAA17 | 10 | 3 |
| Pond | KRK | KRK09 | 9 | 2 |
|  |  | KRK11 | 6 | 2 |
|  |  | KRK16 | 5 | 2 |
|  |  | KRK18 | 4 | 2 |
|  | PYO | PYO01_07_1 | 10 | 3 |
|  |  | PYO01_07_2 | 10 | 1 |
|  |  | PYO06_11_1 | 10 | 1 |
|  |  | PYO09 | 10 | 1 |
|  | RYT | RYT03 | 10 | 2 |
|  |  | RYT12 | 10 | 2 |
|  |  | RYT22 | 10 | 2 |
|  |  | RYT28 | 10 | 2 |
|  | BYN | BYN09 | 6 | 2 |
|  |  | BYN10 | 8 | 2 |
|  |  | BYN16 | 6 | 2 |
|  |  | BYN18 | 6 | 2 |
|  |  | BYN21 | 7 | 2 |
