# Supplementary material for: Recombination rate and efficiency of linked selection in small and large stickleback populations
Source: PLoS Genet. 2026 Oct 28;22(10):e1012335. doi: 10.1371/journal.pgen.1012335 (PMC13716167; doi:10.1371/journal.pgen.1012335)
Supplement: Supplementary file 6 [file pgen.1012335.s005.docx]

### **S4 Table.** Sensitivity analyses of downsampling seven linkage maps to PYO marker density.

| Population | Full SNPs recombination rate | Downsampled to PYO SNP density-level rate (mean of 100 reps; 95% CI) |
| --- | --- | --- |
| KRK | 3.90 | 3.96 (3.91–4.01) |
| POR | 3.81 | 3.86 (3.83–3.92) |
| TVA | 3.85 | 3.85 (3.81–3.89) |
| BYN | 3.76 | 3.79 (3.76–3.83) |
| RYT | 3.71 | 3.75 (3.72–3.79) |
| UME | 3.68 | 3.74 (3.69–3.78) |
| RAA | 3.64 | 3.68 (3.65–3.73) |
| PYO | 4.24 | - |
