# Supplementary material for: Recombination rate and efficiency of linked selection in small and large stickleback populations
Source: PLoS Genet. 2026 Oct 28;22(10):e1012335. doi: 10.1371/journal.pgen.1012335 (PMC13716167; doi:10.1371/journal.pgen.1012335)
Supplement: Supplementary file 7 [file pgen.1012335.s006.docx]

### **S5 Table.** Correlations between nucleotide diversity estimates obtained using Pixy and VCFtools across populations.

| Population | Pearson correlation between Pixy and VCFtools *π* |
| --- | --- |
| BYN | 0.950 |
| KRK | 0.956 |
| POR | 0.953 |
| PYO | 0.848 |
| RAA | 0.950 |
| RYT | 0.926 |
| TVA | 0.954 |
| UME | 0.948 |
