# Supplementary material for: Recombination rate and efficiency of linked selection in small and large stickleback populations
Source: PLoS Genet. 2026 Oct 28;22(10):e1012335. doi: 10.1371/journal.pgen.1012335 (PMC13716167; doi:10.1371/journal.pgen.1012335)
Supplement: Supplementary file 8 [file pgen.1012335.s007.docx]

### **S6 Table.** Associations between Pixy estimated nucleotide diversity and crossover number across populations.

| Population | β₁ | β₀ | R² (%) |
| --- | --- | --- | --- |
| RAA | 6.98 × 10⁻⁵*** | 2.40 × 10⁻³*** | 21.39 |
| TVA | 7.50 × 10⁻⁵*** | 2.32 × 10⁻³*** | 23.55 |
| POR | 6.39 × 10⁻⁵*** | 2.35 × 10⁻³*** | 22.06 |
| UME | 8.09 × 10⁻⁵*** | 2.07 × 10⁻³*** | 28.42 |
| KRK | 1.63 × 10⁻⁵*** | 5.25 × 10⁻⁴*** | 8.87 |
| BYN | 2.89 × 10⁻⁵*** | 6.76 × 10⁻⁴*** | 21.52 |
| RYT | 2.46 × 10⁻⁵*** | 7.28 × 10⁻⁴*** | 15.45 |
| PYO | −1.16 × 10⁻⁶ | 1.64 × 10⁻⁴*** | 0.09 |

### ***P < 0.001.
