# Supplementary material for: Recombination rate and efficiency of linked selection in small and large stickleback populations
Source: PLoS Genet. 2026 Oct 28;22(10):e1012335. doi: 10.1371/journal.pgen.1012335 (PMC13716167; doi:10.1371/journal.pgen.1012335)
Supplement: Supplementary file 11 [file pgen.1012335.s010.docx]

### **S3 Fig. Crossover and chromosomal length. A)** Positive correlation between the physical and the genetic distances for the ten linkage maps across 20 autosomal chromosomes. (Females = orange, Males = green, Pearson’s correlation test with the Bonferroni-adjusted p-values marked. r_p-f_ and r_p-m_ are respectively Pearson’s correlation coefficient for females and males for each population. **B)** The number of crossovers per meiosis per chromosome associated with chromosomal length (Gaussian model conducted in glmmTMB). Significance codes: <0.001: ***, <0.01: **, < 0.05: *.
