# Supplementary material for: Recombination rate and efficiency of linked selection in small and large stickleback populations
Source: PLoS Genet. 2026 Oct 28;22(10):e1012335. doi: 10.1371/journal.pgen.1012335 (PMC13716167; doi:10.1371/journal.pgen.1012335)
Supplement: Supplementary file 12 [file pgen.1012335.s011.docx]

### **S4 Fig.** Genomewide recombination rate (cM/Mb) and nucleotide diversity (***π***) for the two ecotypes (orange bars: female; green bars: male), along with the distribution of CpG content and gene density for the reference genome.
