# Supplementary material for: Recombination rate and efficiency of linked selection in small and large stickleback populations
Source: PLoS Genet. 2026 Oct 28;22(10):e1012335. doi: 10.1371/journal.pgen.1012335 (PMC13716167; doi:10.1371/journal.pgen.1012335)
Supplement: Supplementary file 13 [file pgen.1012335.s012.docx]

### **S5 Fig.** Association between per-1Mb nucleotide diversity (π) and recombination rate (cM/Mb) for the eight populations. Model statistics are summarised in the following table, including estimates of the slope (β₁), intercept (β₀), and the proportion of variance explained (R²) separately for each population.

|  | **β_1_** | **β_0_** | **R^2^** |
| --- | --- | --- | --- |
| **RAA** | 9.97E-05*** | 2.71E-03*** | 32.68% |
| **TVA** | 9.57E-05*** | 2.67E-03*** | 30.57% |
| **POR** | 8.48E-05*** | 2.68E-03*** | 25.92% |
| **UME** | 8.76E-05*** | 2.72E-03*** | 29.39% |
| **KRK** | 2.39E-05*** | 5.63E-04*** | 14.55% |
| **BYN** | 4.16E-05*** | 7.32E-04*** | 32.49% |
| **RYT** | 4.01E-05*** | 7.77E-04*** | 27.64% |
| **PYO** | 2.91E-06 | 4.32E-04*** | 0.22% |
