# Supplementary material for: Recombination rate and efficiency of linked selection in small and large stickleback populations
Source: PLoS Genet. 2026 Oct 28;22(10):e1012335. doi: 10.1371/journal.pgen.1012335 (PMC13716167; doi:10.1371/journal.pgen.1012335)
Supplement: Supplementary file 14 [file pgen.1012335.s013.docx]

### **S6 Fig** Chromosomal number of crossovers in the eight populations.The chromosomes are ordered based on their lengths from the shortest to the longest.
