# Supplementary material for: Recombination rate and efficiency of linked selection in small and large stickleback populations
Source: PLoS Genet. 2026 Oct 28;22(10):e1012335. doi: 10.1371/journal.pgen.1012335 (PMC13716167; doi:10.1371/journal.pgen.1012335)
Supplement: Supplementary file 15 [file pgen.1012335.s014.docx]

### **S7 Fig**. Pearson pairwise comparison of recombination rate at 1 Mb non-overlapping windows between A) two ecotypes and B) two sexes. Each datapoint represents the estimated recombination rate within a 1Mb window for each comparison.
