# Supplementary material for: Recombination rate and efficiency of linked selection in small and large stickleback populations
Source: PLoS Genet. 2026 Oct 28;22(10):e1012335. doi: 10.1371/journal.pgen.1012335 (PMC13716167; doi:10.1371/journal.pgen.1012335)
Supplement: Supplementary file 16 [file pgen.1012335.s015.docx]

### **S8 Fig**. Distribution of recombination hotspots (red) and coldspots (blue). The high repeat regions are shaded in grey. The solid blue and dashed blue lines were respectively the per-window CpG contents and gene density (with the latter divided by 20 for an easier demonstration of the two parameters). The chromosome order is from chr1 to chr21, and the last is chrX where the males showed zero recombination rates in the SDR regions (blue dots in the first half).
