# Supplementary material for: Recombination rate and efficiency of linked selection in small and large stickleback populations
Source: PLoS Genet. 2026 Oct 28;22(10):e1012335. doi: 10.1371/journal.pgen.1012335 (PMC13716167; doi:10.1371/journal.pgen.1012335)
Supplement: Supplementary file 17 [file pgen.1012335.s016.docx]

### **S9 Fig**. A) The principal component analyses of the inversion region on chr19. The circles group the three inversion genotypes 0/0, 0/1 and 1/1 from the left to the right. B) The inversion (*inv.*) on chr19 reduces recombination activity. Above: the per-1Mb crossover count along chr19 for 0/0 (no *inv.*), 0/1 (*inv.* heterozygote) and 1/1 (*inv.* homozygote) states. Below: the percentage of parents in the eight populations carrying the three *inv.* states (population order: TVA, POR, UME, RAA, KRK, PYO, RYT, BYN; *cen.*: centromere).
