# Supplementary material for: Recombination rate and efficiency of linked selection in small and large stickleback populations
Source: PLoS Genet. 2026 Oct 28;22(10):e1012335. doi: 10.1371/journal.pgen.1012335 (PMC13716167; doi:10.1371/journal.pgen.1012335)
Supplement: Supplementary file 18 [file pgen.1012335.s017.docx]

### **S10 Fig**. Manhattan plots of the three GWAS analyses. From outside to inner layer, the plots are respectively the window-sized SNP density, and the negative log10-transformed raw *P*-values for associations in the MLM, GLM, and FarmCPU models, respectively. The dotted red line indicates the genome-wide significance threshold based on the Benjamini-Hochberg False Discovery Rate (FDR < 0.05). B. QQ plots of GWAS models used to identify loci associated with the number of genome-wide crossovers. The genomic inflation factors for the three models range from 0.992 to 1.031. The grey shaded area represents the 95% confidence envelope expected under the null hypothesis of no association. FarmCPU terminated after the first iteration, which is equivalent to the GLM before pseudo QTNs are incorporated; therefore, FarmCPU and GLM p values were identical or nearly identical, and the GLM points are largely hidden underneath the FarmCPU points.
