# Supplementary material for: Recombination rate and efficiency of linked selection in small and large stickleback populations
Source: PLoS Genet. 2026 Oct 28;22(10):e1012335. doi: 10.1371/journal.pgen.1012335 (PMC13716167; doi:10.1371/journal.pgen.1012335)
Supplement: Supplementary file 19 [file pgen.1012335.s018.docx]

### **S11 Fig.** Association between per-1Mb nucleotide diversity and A) CpG contents and B) gene density. Model statistics include the estimated slopes (β) and the proportion of variance explained (R²) for the optimal generalised linear mixed model (Gaussian distribution) constructed in glmmTMB, with population as the random effect.
