# Supplementary material for: Recombination rate and efficiency of linked selection in small and large stickleback populations
Source: PLoS Genet. 2026 Oct 28;22(10):e1012335. doi: 10.1371/journal.pgen.1012335 (PMC13716167; doi:10.1371/journal.pgen.1012335)
Supplement: Supplementary file 20 [file pgen.1012335.s019.docx]

### **S12 Fig.** Significantly higher number of crossovers in PYO than the other populations. A) Number of per-individual crossovers in the most inbred population, PYO, versus the other populations. Comparison of B) sex-averaged recombination rate per chromosome and C) number of per-individual crossovers between the two ecotypes without individuals from PYO.
