# Supplementary material for: Recombination rate and efficiency of linked selection in small and large stickleback populations
Source: PLoS Genet. 2026 Oct 28;22(10):e1012335. doi: 10.1371/journal.pgen.1012335 (PMC13716167; doi:10.1371/journal.pgen.1012335)
Supplement: Supplementary file 21 [file pgen.1012335.s023.docx]

# **Response Letter**

**Manuscript ID:** PGENETICS-D-26-00316R1
**Title:** Recombination rate and efficiency of linked selection in small and large stickleback populations

Dear Dr. White,

Thank you for the opportunity to revise our manuscript. As requested, we have included the nucleotide diversity analysis based on Pixy in the “Supplementary Materials” and cited these results in the revised manuscript.

**Editor comment**

We appreciate the thoughtful responses to the reviewers and revision of your manuscript. There was one response we thought would be beneficial to also include in the final manuscript. Reviewer 1 suggested that the use of VCFtools may lead to distorted estimates of nucleotide diversity (major comment 3), but you showed your estimates were consistent between VCFtools and Pixy. Please include the Pixy results as supplemental and refer to these in the manuscript to strengthen your conclusions about nucleotide diversity.

**Response**

Thank you for this suggestion. We have added the comparison of nucleotide diversity estimates from Pixy and VCFtools in S5 Table and the association between nucleotide diversity estimated using Pixy and crossover number in S6 Table. Both tables are now cited in the revised manuscript.

**Journal Requirements**

1) We have noticed that you have uploaded Supporting Information files (Supplementary_data), but you have not included a complete list of legends. Please add a full list of legends for your Supporting Information files after the references list.

2) We notice that your supplementary figures are uploaded with the file type 'Other'. Please amend the file type to 'Supporting Information'. Please ensure that each Supporting Information file has a legend listed in the manuscript after the references list.

We have added complete legends for S1 Data after the references. During submission, all supplementary figures will be designated as Supporting Information, as requested.
