# Supplementary material for: Recombination rate and efficiency of linked selection in small and large stickleback populations
Source: PLoS Genet. 2026 Oct 28;22(10):e1012335. doi: 10.1371/journal.pgen.1012335 (PMC13716167; doi:10.1371/journal.pgen.1012335)
Supplement: Peer review history

# Peer review: Decision Letter 0

**DOI:** 10.1371/journal.pgen.1012335.r001

**Authors:** Kelly Dyer, Michael White

17 May 2026

PGENETICS-D-26-00316

Recombination rate and efficiency of linked selection in small and large stickleback populations

PLOS Genetics

Dear Dr. Wang,

Thank you for submitting your manuscript to PLOS Genetics. After careful consideration, we feel that it has merit but does not fully meet PLOS Genetics's publication criteria as it currently stands. Therefore, we invite you to submit a revised version of the manuscript that addresses the points raised during the review process.

The reviewers agreed that this is an interesting and well-written study that highlights variation in recombination rate across wild populations. However, both reviewers brought up significant concerns with the PYO population. The freshwater-marine rate differences seem to be largely driven by this outlier and this outlier also seems to have the lowest quality linkage map. The reviewers provide a number of good suggestions to test whether the observed rate difference of PYO is not a technical artifact of the dataset.

Please submit your revised manuscript by Jul 16 2026 11:59PM. If you will need more time than this to complete your revisions, please reply to this message or contact the journal office at plosgenetics@plos.org. When you're ready to submit your revision, log on to https://www.editorialmanager.com/pgenetics/ and select the 'Submissions Needing Revision' folder to locate your manuscript file.

Please include the following items when submitting your revised manuscript:

* A letter that responds to each point raised by the editor and reviewer(s). You should upload this letter as a separate file labeled 'Response to Reviewers'. This file does not need to include responses to any formatting updates and technical items listed in the 'Journal Requirements' section below.

* A marked-up copy of your manuscript that highlights changes made to the original version. You should upload this as a separate file labeled 'Revised Manuscript with Track Changes'.

* An unmarked version of your revised paper without tracked changes. You should upload this as a separate file labeled 'Manuscript'.

If you would like to make changes to your financial disclosure, competing interests statement, or data availability statement, please make these updates within the submission form at the time of resubmission. Guidelines for resubmitting your figure files are available below the reviewer comments at the end of this letter.

As the corresponding author, your ORCID iD is verified in the submission system and will appear in the published article. PLOS supports the use of ORCID, and we encourage all coauthors to register for an ORCID iD and use it as well. Please encourage your coauthors to verify their ORCID iD within the submission system before final acceptance, as unverified ORCID iDs will not appear in the published article. Only the individual author can complete the verification step; PLOS staff cannot verify ORCID iDs on behalf of authors.

We look forward to receiving your revised manuscript.

Kind regards,

Michael A White

Academic Editor

PLOS Genetics

Kelly Dyer

Section Editor

PLOS Genetics

Aimée Dudley

Editor-in-Chief

PLOS Genetics

Anne Goriely

Editor-in-Chief

PLOS Genetics

**Journal Requirements:**

If the reviewer comments include a recommendation to cite specific previously published works, please review and evaluate these publications to determine whether they are relevant and should be cited. There is no requirement to cite these works unless the editor has indicated otherwise.

1) Please ensure that the CRediT author contributions listed for every co-author are completed accurately and in full.

At this stage, the following Authors/Authors require contributions: Hongbo Wang, Chaowei Zhang, Kerry Reid, and Juha Merilä. Please ensure that the full contributions of each author are acknowledged in the "Add/Edit/Remove Authors" section of our submission form.

The list of CRediT author contributions may be found here: https://journals.plos.org/plosgenetics/s/authorship#loc-author-contributions

2) We ask that a manuscript source file is provided at Revision. Please upload your manuscript file as a .doc, .docx, .rtf or .tex. If you are providing a .tex file, please upload it under the item type u2018LaTeX Source Fileu2019 and leave your .pdf version as the item type u2018Manuscriptu2019.

3) Please upload all main figures as separate Figure files in .tif or .eps format. For more information about how to convert and format your figure files please see our guidelines:

https://journals.plos.org/plosgenetics/s/figures

4) The resolution of Figure 1 is very low and somewhat difficult to read. It is important that our Editors and Peer Reviewers are able to read all parts of a submission. Please replace this figure with a higher resolution copy.

5) We have noticed that you have uploaded Supporting Information files, but you have not included a list of legends. Please add a full list of legends for your Supporting Information files after the references list.

6) Some material included in your submission may be copyrighted. According to PLOS’s copyright policy, authors who use figures or other material (e.g., graphics, clipart, maps) from another author or copyright holder must demonstrate or obtain permission to publish this material under the Creative Commons Attribution 4.0 International (CC BY 4.0) License used by PLOS journals. Please closely review the details of PLOS’s copyright requirements here: PLOS Licenses and Copyright. If you need to request permissions from a copyright holder, you may use PLOS's Copyright Content Permission form.

Please respond directly to this email and provide any known details concerning your material's license terms and permissions required for reuse, even if you have not yet obtained copyright permissions or are unsure of your material's copyright compatibility. Once you have responded and addressed all other outstanding technical requirements, you may resubmit your manuscript within Editorial Manager.

Potential Copyright Issues:

i) Figure 1B. Please confirm whether you drew the images / clip-art within the figure panels by hand. If you did not draw the images, please provide (a) a link to the source of the images or icons and their license / terms of use; or (b) written permission from the copyright holder to publish the images or icons under our CC BY 4.0 license. Alternatively, you may replace the images with open source alternatives. See these open source resources you may use to replace images / clip-art:

- https://commons.wikimedia.org

- https://openclipart.org/.

ii) Figure 1A. Please (a) provide a direct link to the base layer of the map (i.e., the country or region border shape) and ensure this is also included in the figure legend; and (b) provide a link to the terms of use / license information for the base layer image or shapefile. We cannot publish proprietary or copyrighted maps (e.g. Google Maps, Mapquest) and the terms of use for your map base layer must be compatible with our CC BY 4.0 license.

Note: if you created the map in a software program like R or ArcGIS, please locate and indicate the source of the basemap shapefile onto which data has been plotted.

If your map was obtained from a copyrighted source please amend the figure so that the base map used is from an openly available source. Alternatively, please provide explicit written permission from the copyright holder granting you the right to publish the material under our CC BY 4.0 license.

If you are unsure whether you can use a map or not, please do reach out and we will be able to help you. The following websites are good examples of where you can source open access or public domain maps:

* U.S. Geological Survey (USGS) - All maps are in the public domain. (http://www.usgs.gov)

* PlaniGlobe - All maps are published under a Creative Commons license so please cite u201cPlaniGlobe, http://www.planiglobe.com, CC BY 2.0u201d in the image credit after the caption. (http://www.planiglobe.com/?lang=enl)

* Natural Earth - All maps are public domain. (http://www.naturalearthdata.com/about/terms-of-use/).

**Reviewers' comments:**

Reviewer's Responses to Questions

**Comments to the Authors:**

**Please note here if the review is uploaded as an attachment.**

Reviewer #1: This manuscript uses pedigree-based linkage maps from four freshwater (“small Ne”) and four marine (“large Ne”) nine-spined stickleback populations to ask two questions: (i) whether the classic positive correlation between nucleotide diversity (π) and recombination rate is weaker in small populations, consistent with reduced efficiency of linked selection, and (ii) whether small populations have evolved higher recombination rates, as predicted by theories of recombination modifiers under directional selection. The authors report that the π–recombination relationship is indeed shallower in freshwater populations (and absent in the most homozygous population, PYO), that PYO has significantly higher crossover counts and recombination rate than the marine populations, and that the GWAS suggests a polygenic basis for crossover count variation.

I liked this paper. The questions are important and the dataset is impressive: eight populations, ten high-density linkage maps, and a telomere-to-telomere reference genome, combined with family-based crosses that sidestep the LD-based confounds common in this literature. The biological questions are well-posed and the overall conclusions point in the right direction.

That said, several aspects of the analysis and interpretation need to be addressed before the headline claims can be accepted. In particular the claim of adaptive evolution of recombination rate in PYO and the polygenic basis of recombination rate variation. My concerns fall into three areas: (1) the two Ne estimators speak to very different timescales, and the authors’ interpretation does not adequately distinguish between them; (2) the “elevated recombination in small populations” signal is effectively an n = 1 inference (PYO) that is also the population most vulnerable to technical inflation of map length; and (3) the nucleotide-diversity and GWAS analyses have methodological choices that should be revisited. Importantly, because this paper is well motivated by theory and the data set itself stands on it own, I do not think these changes (even though they will likely result in the result becoming “negative”) would change the overall scientific value or impact of this work in any sense. Individual points follow.

Major comments

1. The crossover–Ne correlation is being interpreted more strongly than the data support, because the two Ne estimators employed by the authors measure different things. The paper reports a significant negative relationship between crossover count and Ne estimated by MSMC2, but not with CurrentNe2 (p = 0.098; lines 183–184). MSMC2’s harmonic-mean Ne over 2–200 kya substantially predates the ~10 kya freshwater colonization that the authors themselves invoke on line 361. For the freshwater populations, the MSMC2-hm therefore largely reflects marine-ancestral demography, not the small-population conditions that are supposed to have driven recombination rate evolution. The negative crossover–MSMC2 relationship cannot be read as evidence that recombination has evolved in response to current drift, and the non-significance of the CurrentNe2 relationship (which is the Ne metric that actually bears on the authors’ hypothesis) deserves considerably more attention. The current framing in the Abstract, Results and Discussion reads as if both estimators tell the same story; they do not. I think the authors need to (a) state explicitly what timescale MSMC2 covers and what it therefore measures for each ecotype, (b) center the evolutionary interpretation on CurrentNe2 (or on a measure tied to post-colonization drift, e.g. ROH load or current π), and (c) soften or remove language that implies a general Ne effect when only the pre-colonization estimator is significant.

2. The claim of “elevated recombination rate in small populations” rests on a single population. Yet, the freshwater–marine rate difference disappears when PYO is excluded (lines 197–198), the GLMM correlation in Figure 2C is visibly driven by that one point, and the “adaptive evolution of recombination” narrative in the Discussion and Abstract rests essentially on PYO alone. I have a few suggestions for the authors to remedy this issue.

Figure S12 already reports the ecotype comparison of sex-averaged recombination rate and per-individual crossover counts with PYO excluded; this analysis should be brought into the main text and Figure 2, and the text should state clearly that the crossover–Ne signal hinges on this one population.

PYO is a technical outlier in ways that matter for map-length inference: it is the most homozygous population (≈91% of its genome in ROH, line 375), has by far the lowest marker count in its linkage map (158,980, vs. ~3.8 M in the marine maps), and simultaneously has the highest estimated genetic map length (1,815 cM; Table 2). A sparse marker set combined with residual genotyping error is a known cause of map-length inflation in Lep-MAP3. The authors use ROH to argue that recombination in freshwater populations is probably underestimated (lines 376–378), but this logic cuts against the inference of an elevated rate in PYO specifically: if ROH plus sparse markers depress recombination estimates in the other three freshwater populations, why should PYO be exempt, given that it has more ROH and fewer markers than any of them? A sensitivity analysis, e.g., re-estimating map length after down-sampling the marine maps to match PYO’s informative-marker density, masking simulated ROH tracts, or both, would help establish whether the PYO signal is biological or methodological. It would also be helpful to report the number of putative double crossovers in close physical proximity, which is a useful marker of genotyping-error-driven map inflation.

With only one point in the relevant small-Ne region of the parameter space, the “elevated recombination rate evolves in small populations” framing should be presented as a hypothesis generated by these data, not a conclusion supported by them.

3. Nucleotide diversity is estimated without invariant sites, and this could distort the central result. π is estimated with VCFtools on SNPs only (line 494). VCFtools does not account for missing data or for invariant but callable sites, and estimating π this way biases the result, particularly when comparing populations that differ in per-site callability. This is almost certainly the case between the marine parents (very high marker density) and freshwater parents (far fewer informative markers and extensive ROH). Because the π–recombination relationship and the ecotype contrast in π are central results, this matters. I think the authors need to re-estimate π with a method that uses invariant sites and handles missingness explicitly (e.g. pixy) and confirm that (i) the positive π–recombination correlation, (ii) its shallower slope in freshwater, and (iii) the absence of correlation in PYO are all robust to this change. The authors should also report the per-population callable fraction and pairwise differences in callability so the reader can judge the magnitude of the potential bias.

4. The Methods for MSMC2 and CurrentNe2 are deferred to Zhang et al. (2026), a submitted manuscript that is not accessible to the reader (line 506). Even with the Supplementary Materials and Methods in hand, the reader cannot reconstruct these Ne estimates: the mutation rate and generation time assumed for MSMC2, the segment/bin filters, the CurrentNe2 input SNP set and assumed recombination rate, and the minor allele frequency thresholds are all missing. At resubmission, please provide a self-contained Methods description of MSMC2 and CurrentNe2 that does not depend on an unpublished paper.

5. Lines 498–500 state that windows with >20% repeat content were excluded from gene-density and CpG estimation. It is not stated whether this filter was also applied to the π and recombination-rate window analyses. Because repeat content is strongly non-randomly distributed with respect to recombination rate (enriched at telomeres and centromeres), inconsistent masking across analyses could either manufacture or obscure associations. Please state explicitly which analyses used the >20% filter, report how many 1 Mb windows were removed per chromosome, and confirm that the main results are insensitive to alternative repeat-content thresholds (e.g., 10%, 30%, or a gradient).

6. The GWAS is run on the wild-caught F0 parents — a total of 80 individuals (Table 1) distributed across eight populations. This is modest power for mapping a quantitative trait with a genome-wide test. Across the three methods, only three QTL survive FDR < 0.05 in FarmCPU and GLM, and zero survive in MLM. The “48 suggestive QTL” from FarmCPU are based on a nominal p < 1e-05 cutoff without genome-wide correction, and the between-method concordance (MLM 4, GLM 48, FarmCPU 48) is not reassuring. These data do not support the current wording of “GWAS analyses suggested a polygenic basis for recombination rates” (Abstract) and especially not the Author Summary statement that “recombination is not controlled by a single gene but is a complex trait influenced by many genes in different parts of the genome.” I think the authors should (a) report QQ plots, λGC for all three methods (Manhattan plots are already in Figure S10 of the supplement); (b) report the per-method set overlap of significant hits; (c) state the sample size and expected statistical power; and (d) soften the polygenicity language to reflect the FDR-corrected results.

7. The Ne values reported in the Abstract do not match Table 1. The Abstract gives mean Ne ≈ 2,578 for freshwater and 86,742 for marine populations, but neither value corresponds to the mean of either Ne estimator in Table 1. For the marine populations, the mean of NeMSMC2-hm is ≈264,925 and the mean of NeCurrentNe2 is ≈14,324. I cannot reconstruct how 2,578 or 86,742 were obtained from Table 1. Please state in the Abstract, Table 1 legend, and Methods exactly what Ne estimator and summary statistic are being reported, and reconcile the numbers. A range across the four populations of each ecotype would perhaps be more informative than a point estimate.

Minor comments

9. Lines 561–563 duplicate lines 558–559 almost verbatim on FarmCPU controlling for population stratification. Please remove the duplicate. Similar near-duplicate phrasing also appears in the Discussion.

10. Line 417 cites “Zhang et al. 2025” whereas every other citation of that reference in the manuscript is to “Zhang et al. 2026” (e.g., lines 121, 402, 497, 506, 512).

11. Hotspot (top 5% of windows) and coldspot (zero crossovers) are operational cutoffs, not biological categories, and are sensitive to map resolution. Given mean per-chromosome crossover counts of ~1.84 per meiosis, a 1 Mb window with zero observed crossovers is often a sampling artifact rather than a biological cold spot. Please either demonstrate robustness to alternative cutoffs (e.g., top 1% / 10%) or frame the dichotomy as operational.

12. Figure S9 already reports inversion genotype frequencies across populations and per-genotype recombination on chr19. Please reference Figure S9 when the chr19 inversion is raised in the Discussion, and state explicitly in the main text whether the inversion region was included in or masked from the genome-wide rate estimates.

13. Table 1 legend defines NeMSMC2-hm as the harmonic mean over 2–200 kya, but the main text introduces “past Ne estimated by MSMC2” (line 183) without this timescale. Please state the timescale the first time the estimator is used in the text.

14. Please ensure the GitHub repository (https://github.com/LeoHongboWANG/Recombination_in_Sticklebacks) includes a README describing script dependencies, input-file expectations, and how to reproduce the main figures. I did not evaluate the code.

15. Typos: line 121–122 “To enhance the accuracy genome-wide…” is missing “of”; line 72 has an extra space before the period; line 399 refers to “methylation levels” where “CpG content” is what is actually measured; I think “Varandharjan” on line 480 is a misspelling of Varadharajan.

Reviewer #2: I thought this manuscript was interesting and well-written. Few studies have examined patterns of variation in recombination rate for multiple wild populations of the same species, so this study is very useful. The paper also provides interesting insight into the relationship between nucleotide diversity, recombination rate and effective population. Lastly, It identifies candidate genes/regions contributing to variance in recombination rate.

However, I had two primary issues: the first was with the emphasis that the PYO population had the smallest Ne and the highest recombination rate. Given that this population appeared as an outlier it was not reassuring that it had the lowest quality linkage map, with substantially fewer markers. Could the recombination rate be inflated by the low marker density? Could you down sample the other populations to the same density to ensure it is not an artefact? Were there also fewer markers for this population for the estimation of Ne? If so, again could this be driving the pattern? A considerable fraction of the discussion and angle of the paper is focused on this exceptional (outlier) population so I think there needs to be more reassurance in the validity of this result. The second is that the observed recombination rate difference between the ecotypes could be due to differences in genome size and structure differences. This is a fairly large caveat and should be more thoroughly addressed. Is there a freshwater reference that could be used to check for this potential effect? Is there another way to address this potentially fundamental constraint of the current dataset?

**********

**Have all data underlying the figures and results presented in the manuscript been provided?**

Large-scale datasets should be made available via a public repository as described in the *PLOS Genetics*  data availability policy, and numerical data that underlies graphs or summary statistics should be provided in spreadsheet form as supporting information.

Reviewer #1: Yes

Reviewer #2: Yes

**********

PLOS authors have the option to publish the peer review history of their article (what does this mean?). If published, this will include your full peer review and any attached files.

If you choose “no”, your identity will remain anonymous but your review may still be made public.

**Do you want your identity to be public for this peer review?**  For information about this choice, including consent withdrawal, please see our Privacy Policy.

Reviewer #1: No

Reviewer #2: No

**Figure resubmission:**

While revising your submission, we strongly recommend that you use PLOS’s NAAS tool (https://ngplosjournals.pagemajik.ai/artanalysis) to test your figure files. NAAS can convert your figure files to the TIFF file type and meet basic requirements (such as print size, resolution), or provide you with a report on issues that do not meet our requirements and that NAAS cannot fix.

After uploading your figures to PLOS’s NAAS tool - https://ngplosjournals.pagemajik.ai/artanalysis, NAAS will process the files provided and display the results in the "Uploaded Files" section of the page as the processing is complete. If the uploaded figures meet our requirements (or NAAS is able to fix the files to meet our requirements), the figure will be marked as "fixed" above. If NAAS is unable to fix the files, a red "failed" label will appear above. When NAAS has confirmed that the figure files meet our requirements, please download the file via the download option, and include these NAAS processed figure files when submitting your revised manuscript.

**Reproducibility:**

To enhance the reproducibility of your results, we recommend that authors of applicable studies deposit laboratory protocols in protocols.io, where a protocol can be assigned its own identifier (DOI) such that it can be cited independently in the future. Additionally, PLOS ONE offers an option to publish peer-reviewed clinical study protocols. Read more information on sharing protocols at https://plos.org/protocols?utm_medium=editorial-email&utm_source=authorletters&utm_campaign=protocols

# Author comment: Author response to Decision Letter 1

**DOI:** 10.1371/journal.pgen.1012335.r002

6 Aug 2026

Attachment Submitted filename: Response_Letter_PG.pdf

# Peer review: Decision Letter 1

**DOI:** 10.1371/journal.pgen.1012335.r003

**Authors:** Kelly Dyer, Michael White

15 Sep 2026

PGENETICS-D-26-00316R1

Recombination rate and efficiency of linked selection in small and large stickleback populations

PLOS Genetics

Dear Dr. Wang,

Thank you for submitting your manuscript to PLOS Genetics. After careful consideration, we feel that it has merit but does not fully meet PLOS Genetics's publication criteria as it currently stands. Therefore, we invite you to submit a revised version of the manuscript that addresses the points raised during the review process.

We appreciate the thoughtful responses to the reviewers and revision of your manuscript. There was one response we thought would be beneficial to also include in the final manuscript. Reviewer 1 suggested that the use of VCFtools may lead to distorted estimates of nucleotide diversity (major comment 3), but you showed your estimates were consistent between VCFtools and Pixy. Please include the Pixy results as supplemental and refer to these in the manuscript to strengthen your conclusions about nucleotide diversity.

Please submit your revised manuscript within by . If you will need more time than this to complete your revisions, please reply to this message or contact the journal office at plosgenetics@plos.org. When you're ready to submit your revision, log on to https://www.editorialmanager.com/pgenetics/ and select the 'Submissions Needing Revision' folder to locate your manuscript file.

Please include the following items when submitting your revised manuscript:

* A letter that responds to each point raised by the editor and reviewer(s). You should upload this letter as a separate file labeled 'Response to Reviewers'. This file does not need to include responses to formatting updates and technical items listed in the 'Journal Requirements' section below.

* A marked-up copy of your manuscript that highlights changes made to the original version. You should upload this as a separate file labeled 'Revised Manuscript with Track Changes'.

* An unmarked version of your revised paper without tracked changes. You should upload this as a separate file labeled 'Manuscript'.

If you would like to make changes to your financial disclosure, competing interests statement, or data availability statement, please make these updates within the submission form at the time of resubmission. Guidelines for resubmitting your figure files are available below the reviewer comments at the end of this letter.

As the corresponding author, your ORCID iD is verified in the submission system and will appear in the published article. PLOS supports the use of ORCID, and we encourage all coauthors to register for an ORCID iD and use it as well. Please encourage your coauthors to verify their ORCID iD within the submission system before final acceptance, as unverified ORCID iDs will not appear in the published article. Only the individual author can complete the verification step; PLOS staff cannot verify ORCID iDs on behalf of authors.

We look forward to receiving your revised manuscript.

Kind regards,

Michael A White

Academic Editor

PLOS Genetics

Kelly Dyer

Section Editor

PLOS Genetics

Aimée Dudley

Editor-in-Chief

PLOS Genetics

Anne Goriely

Editor-in-Chief

PLOS Genetics

**Journal Requirements:**

1) We have noticed that you have uploaded Supporting Information files (Supplementary_data), but you have not included a complete list of legends. Please add a full list of legends for your Supporting Information files after the references list.

2) We notice that your supplementary figures are uploaded with the file type 'Other'. Please amend the file type to 'Supporting Information'. Please ensure that each Supporting Information file has a legend listed in the manuscript after the references list.

If the reviewer comments include a recommendation to cite specific previously published works, please review and evaluate these publications to determine whether they are relevant and should be cited. There is no requirement to cite these works unless the editor has indicated otherwise.

[Note: HTML markup is below. Please do not edit.]

**Reviewers' comments:**

Reviewer's Responses to Questions

**Comments to the Authors:**

**Please note here if the review is uploaded as an attachment.**

Reviewer #2: I am very pleased with the thoughtful and careful revision undertaken by the authors. All of my original comments (and those of the other reviewer) were thoroughly addressed and in my read through of the revision I did not encounter any new issues.

**********

**Have all data underlying the figures and results presented in the manuscript been provided?**

Large-scale datasets should be made available via a public repository as described in the *PLOS Genetics*  data availability policy, and numerical data that underlies graphs or summary statistics should be provided in spreadsheet form as supporting information.

Reviewer #2: Yes

**********

PLOS authors have the option to publish the peer review history of their article (what does this mean?). If published, this will include your full peer review and any attached files.

If you choose “no”, your identity will remain anonymous but your review may still be made public.

**Do you want your identity to be public for this peer review?**  For information about this choice, including consent withdrawal, please see our Privacy Policy.

Reviewer #2: No

[NOTE: If reviewer comments were submitted as an attachment file, they will be attached to this email and accessible via the submission site. Please log into your account, locate the manuscript record, and check for the action link "View Attachments". If this link does not appear, there are no attachment files.]

**Figure resubmission:**

**Reproducibility:**

To enhance the reproducibility of your results, we recommend that authors deposit laboratory protocols in protocols.io, where a protocol can be assigned its own identifier (DOI) such that it can be cited independently in the future. Additionally, PLOS ONE offers an option to publish peer-reviewed clinical study protocols. Read more information on sharing protocols at https://plos.org/protocols?utm_medium=editorial-email&utm_source=authorletters&utm_campaign=protocols

# Author comment: Author response to Decision Letter 2

**DOI:** 10.1371/journal.pgen.1012335.r004

27 Sep 2026

Attachment Submitted filename: Response-Letter-R2-v1.docx

# Editor report: Decision Letter 2

**DOI:** 10.1371/journal.pgen.1012335.r005

**Authors:** Kelly Dyer, Michael White

30 Sep 2026

Dear Dr Wang,

We are pleased to inform you that your manuscript entitled "Recombination rate and efficiency of linked selection in small and large stickleback populations" has been editorially accepted for publication in PLOS Genetics. Congratulations!

Before your submission can be formally accepted and sent to production you will need to complete our formatting changes, which you will receive in a follow up email. Please be aware that it may take several days for you to receive this email; during this time no action is required by you. Please note: the accept date on your published article will reflect the date of this provisional acceptance, but your manuscript will not be scheduled for publication until the required changes have been made.

Once your paper is formally accepted, an uncorrected proof of your manuscript will be published online ahead of the final version, unless you’ve already opted out via the online submission form. If, for any reason, you do not want an earlier version of your manuscript published online or are unsure if you have already indicated as such, please let the journal staff know immediately at plosgenetics@plos.org.

In the meantime, please log into Editorial Manager at https://www.editorialmanager.com/pgenetics/, click the "Update My Information" link at the top of the page, and update your user information to ensure an efficient production and billing process. Note that PLOS requires an ORCID iD for all corresponding authors. Therefore, please ensure that you have an ORCID iD and that it is validated in Editorial Manager. To do this, go to ‘Update my Information’ (in the upper left-hand corner of the main menu), and click on the Fetch/Validate link next to the ORCID field.  This will take you to the ORCID site and allow you to create a new iD or authenticate a pre-existing iD in Editorial Manager.

If you have a press-related query, or would like to know about making your underlying data available (as you will be aware, this is required for publication), please see the end of this email. If your institution or institutions have a press office, please notify them about your upcoming article at this point, to enable them to help maximise its impact. Inform journal staff as soon as possible if you are preparing a press release for your article and need a publication date.

Thank you again for supporting open-access publishing; we are looking forward to publishing your work in PLOS Genetics!

Yours sincerely,

Michael A White

Academic Editor

PLOS Genetics

Kelly Dyer

Section Editor

PLOS Genetics

Aimée Dudley

Editor-in-Chief

PLOS Genetics

Anne Goriely

Editor-in-Chief

PLOS Genetics

www.plosgenetics.org

BlueSky: @plos.bsky.social

----------------------------------------------------

Comments from the reviewers (if applicable):

----------------------------------------------------

**Data Deposition**

If you have submitted a Research Article or Front Matter that has associated data that are not suitable for deposition in a subject-specific public repository (such as GenBank or ArrayExpress), one way to make that data available is to deposit it in the Dryad Digital Repository. As you may recall, we ask all authors to agree to make data available; this is one way to achieve that. A full list of recommended repositories can be found on our website.

The following link will take you to the Dryad record for your article, so you won't have to re‐enter its bibliographic information, and can upload your files directly:

http://datadryad.org/submit?journalID=pgenetics&manu=PGENETICS-D-26-00316R2

More information about depositing data in Dryad is available at http://www.datadryad.org/depositing. If you experience any difficulties in submitting your data, please contact help@datadryad.org for support.

Additionally, please be aware that our data availability policy requires that all numerical data underlying display items are included with the submission, and you will need to provide this before we can formally accept your manuscript, if not already present.

----------------------------------------------------

**Press Queries**

If you or your institution will be preparing press materials for this manuscript, or if you need to know your paper's publication date for media purposes, please inform the journal staff as soon as possible so that your submission can be scheduled accordingly. Your manuscript will remain under a strict press embargo until the publication date and time. This means an early version of your manuscript will not be published ahead of your final version. PLOS Genetics may also choose to issue a press release for your article. If there's anything the journal should know or you'd like more information, please get in touch via plosgenetics@plos.org.

# Editor report: Acceptance letter

**DOI:** 10.1371/journal.pgen.1012335.r006

**Authors:** Kelly Dyer, Michael White

PGENETICS-D-26-00316R2

Recombination rate and efficiency of linked selection in small and large stickleback populations

Dear Dr Wang,

We are pleased to inform you that your manuscript entitled "Recombination rate and efficiency of linked selection in small and large stickleback populations" has been formally accepted for publication in PLOS Genetics! Your manuscript is now with our production department and you will be notified of the publication date in due course.

The corresponding author will soon be receiving a typeset proof for review, to ensure errors have not been introduced during production. Please review the PDF proof of your manuscript carefully, as this is the last chance to correct any errors. Please note that major changes, or those which affect the scientific understanding of the work, will likely cause delays to the publication date of your manuscript.

Soon after your final files are uploaded, unless you have opted out or your manuscript is a front-matter piece, the early version of your manuscript will be published online. The date of the early version will be your article's publication date. The final article will be published to the same URL, and all versions of the paper will be accessible to readers.

For Research Articles, you will receive an invoice from PLOS for your publication fee after your manuscript has reached the completed accept phase. If you receive an email requesting payment before acceptance or for any other service, this may be a phishing scheme. Learn how to identify phishing emails and protect your accounts at https://explore.plos.org/phishing.

Thank you again for supporting PLOS Genetics and open-access publishing. We are looking forward to publishing your work!

With kind regards,

Janani Seenivasan

PLOS Genetics

On behalf of:

The PLOS Genetics Team

Carlyle House, Carlyle Road, Cambridge CB4 3DN | United Kingdom

plosgenetics@plos.org | +44 (0) 1223-442823

plosgenetics.org | Twitter: @PLOSGenetics
